# Cordycepin‐induced unfolded protein response‐dependent cell death, and AKT/MAPK‐mediated drug resistance in mouse testicular tumor cells

**DOI:** 10.1002/cam4.2285

**Published:** 2019-05-30

**Authors:** Ming‐Min Chang, Bo‐Syong Pan, Chia‐Yih Wang, Bu‐Miin Huang

**Affiliations:** ^1^ Department of Cell Biology and Anatomy, College of Medicine National Cheng Kung University Tainan Taiwan, Republic of China; ^2^ Department of Medical Research China Medical University Hospital, China Medical University Taichung Taiwan, Republic of China; ^3^ Present address: Department of Cancer Biology Wake Forest University School of Medicine Winston Salem North Carolina USA

**Keywords:** cordycepin, drug resistance, MA‐10 mouse Leydig tumor cell, PERK‐eIF2α/IRE1‐XBP1 pathways, testicular cancer, unfolded protein response

## Abstract

Testicular cancer is the most commonly diagnosed cancer in men at 15‐44 years of age, and radical orchidectomy combined with chemotherapy is currently considered as the standard treatment. However, drugs resistance and side effects that impact the quality of life for patients with testicular cancer have not been markedly improved in recent decades. In this study, we characterized the pharmacological exacerbation of the unfolded protein response (UPR), which is an effective approach to kill testicular cancer cells, by carrying out a clustering analysis of mRNA expression profiles and the immunobloting examination of cordycepin‐treated MA‐10 cells. The UPR is executed in response to endoplasmic reticulum stress to complement by an apoptotic response if the defect cannot be resolved. Results showed that cordycepin significantly modulated FoxO/P15/P27, PERK‐eIF2α (apoptotic), and the IRE1‐XBP1 (adaptive) UPR pathways. Interestingly, a fraction of MA‐10 cells survived after cordycepin treatment, the AKT, LC3 I/II, and MAPK signaling pathways were highly induced in attached cells as compared to the suspended cells, illustrating the drug resistance to cordycepin via activating AKT and MAPK pathways in MA‐10 cells. In summary, PERK‐eIF2α signaling pathway is required for pro‐apoptotic UPR in MA‐10 cell death following cordycepin treatment, suggesting a potential therapeutic application in treating testicular cancer. However, activation of AKT and MAPK pathways could possibly result in drug resistance to cordycepin in MA‐10 cells.

## 1 INTRODUCTION

Testicular cancer, including germ cell, Sertoli cell, and Leydig cell tumors, is one of the most common solid tumors in men between 15 and 44 years of age with about 9310 new cases found in the United States annually.^1^, ^2^ Chemotherapy with or without surgery and/or radiotherapy combination is an important therapeutic strategy for testicular cancers.^3^, ^4^ However, the clinical applications of chemotherapy are limited because of severe side effects, such as cardiovascular diseases and respiratory infections, which lead to a 6% increased risks of noncancer death in long‐term testicular cancer survivors after treatments with chemotherapy and/or radiotherapy.^5^, ^6^ This necessitates the discovery and development of new chemotherapeutic agents that effectively inhibit testicular cancer with minimized off‐target side effects.

The endoplasmic reticulum (ER) is a multifunctional organelle responsible for the synthesis, proper folding and assembly of newly synthesized exportable proteins, lipid and sterol biosynthesis, carbohydrate metabolism, and free calcium storage.^7^, ^8^ ER homeostasis can be disrupted by physiological and pathological stimuli resulting an accumulation of unfolded or misfolded proteins, a condition known as ER stress.^8^, ^9^ ER stress then subsequently triggers the unfolded protein response (UPR), a complex intracellular signal transduction pathway, to increase the protein folding capacity and decrease unfolded protein load.^8^, ^9^, ^10^ In response to the environmental and genetic stresses, the UPR and autophagy are important mechanisms involved in the regulation of cellular stress responses, and both of them are interconnected.^10^, ^11^ Many studies have focused on the UPR and autophagy as novel therapeutic targets for cancers because of the difference of metabolic mechanism and dependence on stress responses between normal and malignant cells.^12^, ^13^, ^14^ ER stress induces UPR by activating three master sensors located in the ER membrane: double‐stranded RNA‐activated protein kinase (PKR)‐like ER kinase (PERK), activating transcription factor 6 (ATF6), and inositol‐requiring enzyme 1 (IRE1).^15^, ^16^, ^17^ First, activation of PERK could result in the phosphorylation of eukaryotic translation initiation factor‐2a and translation of the transcription factor ATF4, resulting in blocking protein translation and reducing the protein burden within the ER, cell cycle arrest, and thereby preventing further damage to the cells. Second, ATF6, after cleavage in the Golgi, translocates to the nucleus and activates the transcription of genes involved in protein folding, processing, and degradation, such as ATF4, CHOP, and x‐box‐binding protein (XBP1). Third, IRE1‐α mediates splicing of XBP1 increases transcription of ER‐resident chaperones of the protein degradation machinery.^15^, ^16^, ^17^

**Figure 1:**
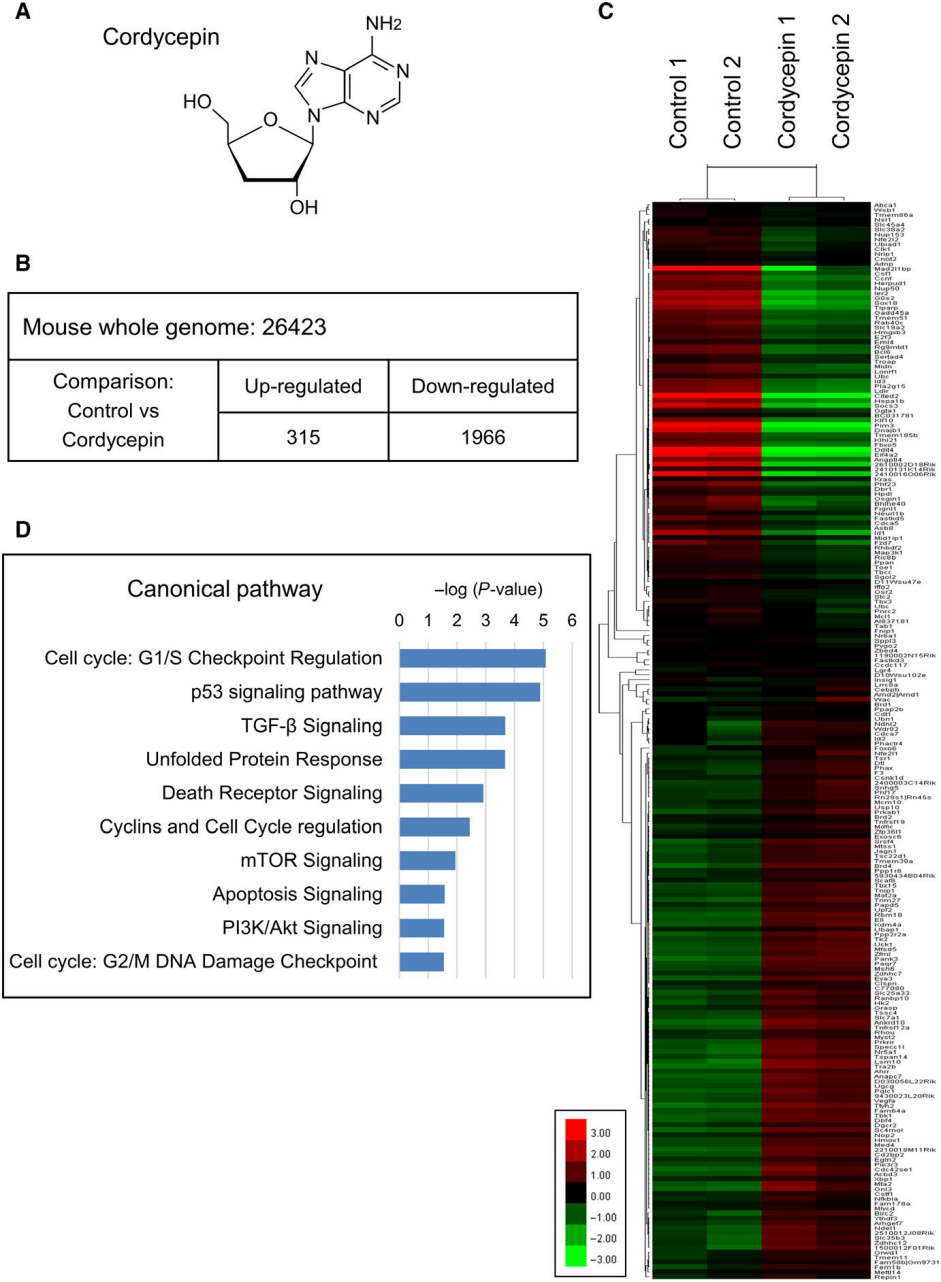
Gene ontology analysis for cordycepin regulated genes in MA‐10 cells. Microarray analysis of MA‐10 cell gene expression in response to 100 μmol/L cordycepin treatment for 3 h. A, The chemical structure of Cordycepin. B, The numbers of up‐regulated and down‐regulated mRNA in MA‐10 cells were altered more than twofold in response to cordycepin treatment identified for gene ontology analysis. C, A clustered heat map represents the genes with most variable expression in response to cordycepin treatment. The values of gene expression fold change were transformed to log_2_‐ratio (log_2_ fold change). Red and green colors indicate up‐ and down‐regulated gene expression, respectively, and intermediate values will be colored according to the gradient. Trees on the left are gene clusters. D, Top 10 cell death/survival‐related canonical pathways ranked by −log (*P*‐value) were revealed by IPA core analysis. IPA, Ingenuity^®^ Pathway Analysis

Cordycepin (3’‐deoxyadenosine; C_10_H_13_N_5_O_3_) (Figure 1A), a major bioactive compound of *Cordyceps sinensis*, has a wide range of biological effects regulating steroidogenesis, inflammation, and platelet aggregation.^18^, ^19^, ^20^, ^21^ Besides, studies have shown that cordycepin exerts a large variety of antitumor abilities.^22^, ^23^ However, it is rare on the investigation of antitumor effect of cordycepin on testicular tumor. Previously, we have reported that cordycepin could induce MA‐10 cell apoptosis by activating p38 MAPKs and inhibiting PI3K/AKT signaling pathways.^24^ However, the downstream signaling consequence of cordycepin‐induced p38 activation still needs to be further revealed. Moreover, in the present study, we found that some cells survive after cordycepin treatments, indicating that there were different populations in MA‐10 cells, some of them were resistance to cordycepin. Drug resistance has been demonstrated in numerous tumor treatments, and several different mechanisms have been investigated and revealed.^25^ However, the possible mechanisms related to cordycepin‐induced drug resistance in MA‐10 cells remain elusive, which is also investigated in this study.

## 2 MATERIALS AND METHODS

### 2.1 Cell culture

The mouse Leydig tumor cell line, MA‐10, was a gift from Dr Mario Ascoli (University of Iowa, Iowa City, IA, USA).^26^ MA‐10 cells were maintained in Waymouth MB 752/1 medium containing 20 mmol/L HEPES, 1.12 g/L NaHCO_3_ and 10% fetal bovine serum and incubated at 37°C in 5% CO_2_ atmosphere. The 6 × 10^5^ MA‐10 cells were seed in 6‐cm dishes 18 hours prior to the initiation of the treatment and then treated with different concentrations of cordycepin (10, 50, 100, 500, and 1000 μmol/L), taxol (100 and 1000 nmol/L) or cisplatin (100 and 200 μmol/L) or plain media (negative control) for 12 or 24 hours, respectively. Taxol‐ and cisplatin‐treated MA‐10 cells were used as a positive control. All chemicals and materials used in this study are listed in Table S1.

### 2.2 Microarray analysis

For transcript profiling of cordycepin‐treated MA‐10 cells, mouse whole‐genome microarrays were performed on a Mouse OneArray^®^ Version 2.0 platform (Phalanx Biotech Group, Taiwan). The microarray data are available at https://www.ncbi.nlm.nih.gov/geo/query/acc.cgi?acc=GSE112513. Total RNA was extracted from MA‐10 cells treated with 100 μmol/L cordycepin or DMSO (vehicle control) for 3 hours. Each group had duplicates. Microarray analysis was conducted according to the manufacturer's protocol. Genes that were significantly up‐ or down‐regulated by more than twofold were subjected to GO enrichment analysis using the Database for Annotation, Visualization, and Integrated Discovery tools (http://david.abcc.ncifcrf.gov/). Normalized intensities were transformed into gene expression log_2_ ratios between the control and treatment groups. The genes with log_2_ ratio ≥1 or log_2_ ratio ≤ −1 and *P* < 0.05 were tested for further analysis. The Ingenuity^®^ Pathway Analysis (IPA^®^, QIAGEN Bioinformatics) program was used to perform the pathway estimation and directional effect prediction.

### 2.3 Western blotting

Treated MA‐10 cells were rinsed with ice‐cold PBS and harvested using lysis buffer (20 mmol/L Tris‐base, 150 mmol/L NaCl, 1 mmol/L EDTA, 1 mmol/L EGTA, 1% Triton X‐100, 2.5 mmol/L sodium pyrophosphate, 1 mmol/L β‐glycerophosphate, and 1 mmol/L Na_3_VO_4_). The lysate was centrifuged (12 000 *g*, 12 minutes, 4°C), and the supernatant was collected. Thirty‐five micrograms of total proteins was separated by 12.5% sSDS‐PAGE and transferred to PVDF membranes. The membranes were blocked with tris‐buffered saline containing 0.1% Tween‐20 and 5% nonfat dry milk at room temperature for 1 hour and then blotted with specific primary antibodies at 4°C for overnight. The signal was detected with horseradish peroxidase (HRP)‐conjugated secondary antibody and visualized with an enhanced chemiluminescent HRP substrate. The protein levels were quantitated by a computer‐assisted image analysis system (UVP bioImage system software, UVP Inc, USA). β‐actin served as a loading control. The integrated optical density of the proteins was normalized to β‐actin in each lane. All antibodies used in this study are listed in Table S2.

### 2.4 Morphological observation

After the treatment with 100 μmol/L cordycepin, 100 μmol/L taxol, 100 μmol/L cisplatin for 24 hours, suspended and attached MA‐10 cells were collected separately and then recultured for 72 hours more (96 hours after treatment). MA‐10 cells were examined for morphological changes at 24 and 96 hours after treatments under Olympus CK40 light microscopy and recorded images by Olympus DP20 digital camera (Olympus, Tokyo, Japan).

### 2.5 Cell cycle analysis

Treated MA‐10 cells were harvested by trypsin digestion, centrifugation, and then washed by isoton II and fixed by 70% ethanol for at least 2 hours at −20°C. After fixation, cells were washed with cold isoton II and then collected by centrifugation. Cell suspensions were mixed with 100 μg/mL RNase and stained with 40 μg/mL propidium iodine (PI) solution for 30 minutes. The stained cells were analyzed at *λ* = 488nm for PI detection by BD FACScan flow cytometer (Becton‐Dickinson, Mountain View, CA, USA). Cells in subG1 phase have less DNA contents on cell cycle distribution, which is considered to be DNA fragmentation and as an outcome of cell apoptosis.

### 2.6 Statistical analysis

Statistically significant differences between groups were determined using Student's *t* test, one‐way analysis of variance or the least significant difference test. The *P* < 0.05 were considered to be statistically significant in this study.

## 3 RESULTS

### 3.1 Clustering analysis of mRNA expression profiles in cordycepin‐treated MA‐10 cells

In order to identify the genes potentially involved in the anti‐cancer activity of cordycepin, microarray analysis of MA‐10 mouse Leydig tumor cells treated with 100 μmol/L cordycepin or DMSO (vehicle control) for 3 hours was conducted. Among the 26 423 mouse whole genome tested, 315 and 1966 genes were significantly up‐ and down‐regulated more than twofold by cordycepin, respectively (Figure 1B). The genes with most variable expression in response to cordycepin treatment were visualized by clustering heatmap (Figure 1C). The top 10 cell death/survival‐related canonical pathways, analyzed by IPA^®^, were mainly involved in cell cycle checkpoint regulation, p53 signaling, UPR, death receptor signaling, mTOR signaling, apoptosis signaling, and PI3K/Akt signaling (Figure 1D).

**Figure 2:**
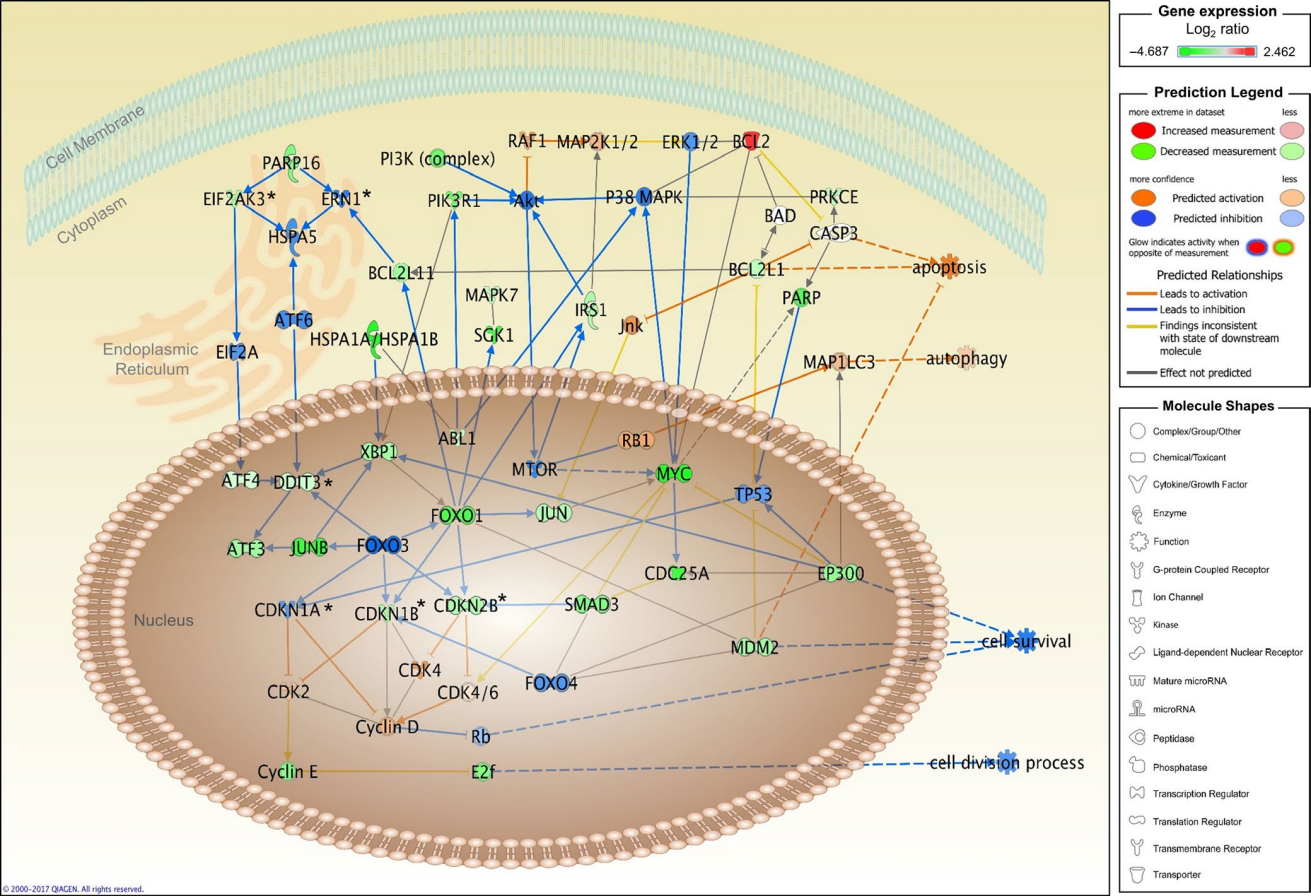
The directional effect predictions of the highly relevant signal pathways affected by cordycepin in MA‐10 cells. The genes significantly regulated by cordycepin were mapped onto the signaling network of the top 10 cell death/survival‐related canonical pathways described in Figure 1D. The activation or inhibition effects of path were predicted by IPA molecule activity predictor (MAP) overlay tools based on our microarray finding. Red‐Green color indicates the level of gene expression. Orange color indicates the activation effect, whereas blue color indicates inhibition effect. Yellow color means that the finding is inconsistent with the state of downstream molecule, and gray color means no prediction on the path. *The synonyms of genes: EIF2AK3: PERK, ERN1: IRE1, MAP1LC3: LC3, DDIT3: CHOP, CDKN1A: p21, CDKN1B: p27, CDKN2B: p15

To further identify the genes potentially involved in cell death induced by cordycepin, the IPA^®^ molecule activity predictor (IPA‐MAP) tool was used to predict the activation or inhibition effects of signaling pathways in the cordycepin‐treated MA‐10 cells. First, the microarray results showed that the expressions of FoxO1, MDM2, c‐Myc, p15 (CDKN2B), p27 (CDKN1B), and cycline E, which are the cell cycle G1/S checkpoint pathway genes, decreased in the cordycepin‐treated group. Second, the IPA‐MAP predictions showed that the FoxO/P15/P27/CDK4 signaling pathways were inhibited (Figure 2). In the UPR pathway, PERK/eIF2α/ATF3/CHOP (apoptosis) and the IRE1/XBP1 (protein refolding) were predicted to be inhibited after cordycepin treatment (Figure 2). Furthermore, the apoptosis and autophagy‐related proteins were activated after cordycepin treatment in the mTOR signaling pathway (Figure 2).

### 3.2 Cordycepin‐induced cell cycle arrest in MA‐10 cells

**Figure 3:**
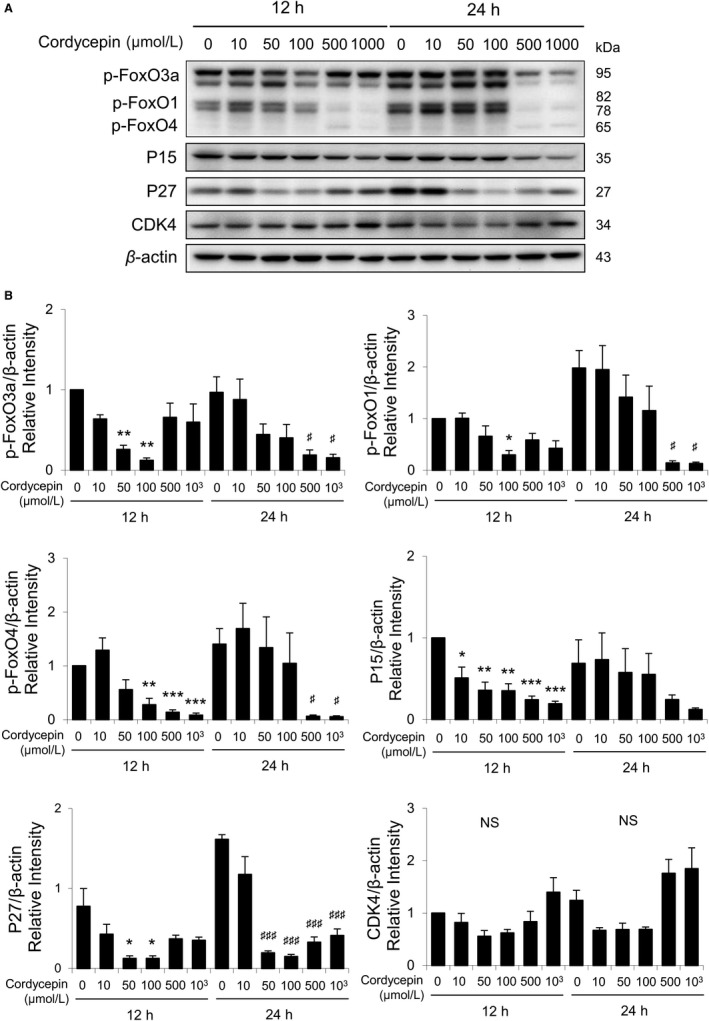
Cordycepin‐induced apoptosis of MA‐10 cells by regulating FoxO signaling pathways. A, Western blot analysis for the expression of phosphorylated FoxO3a, FoxO1, FoxO4, P15, P27, and CDK4 in MA‐10 cells treated with 0, 10, 50, 100, 500, or 1000 μmol/L cordycepin for 12 or 24 h, respectively. B, Quantification of bar graphs shows the integrated optical densities (IOD) of phosphor‐FoxO3a, FoxO1, FoxO4, P15, P27, and CDK4, which were normalized with β‐actin in each lane. All values are represented as the mean ± SEM of three separate experiments. The *P*‐values were calculated using one‐way ANOVA with Tukey's multiple comparisons post‐tests; * or ^#^ *P* < 0.05, ** or ^##^ *P* < 0.01, and *** or ^###^ *P* < 0.001 vs the control group (0 μmol/L cordycepin) at 12 or 24 h, respectively. ANOVA, analysis of variance

To further investigate how cordycepin regulated FoxO/P15/P27/CDK4 pathways, MA‐10 cells were treated with different concentration of cordycepin (0, 10, 50, 100, 500, or 1000 μmol/L) for 12 and 24 hours, respectively, and the expressions of phosphor (p)‐FoxO3a, p‐FoxO1, p‐FoxO4, P15, P27, and CDK4 were detected by Western blotting. Results showed that expression of phosphor‐FoxO3a at 12 hours of cordycepin treatment gradually decreased from 0 to 100 μmol/L, while it was induced by 500 and 1000 μmol/L cordycepin (Figure 3A,B). However, expression of phosphor‐FoxO3a at 24 hours of cordycepin treatment gradually decreased from 0 to 1000 μmol/L (Figure 3B). The expressions of phosphor‐FoxO1, phosphor‐FoxO4, P15, and P27 were decreased dose dependently after 12‐ and 24‐hour cordycepin exposure (Figure 3A,B). Although there is no statistical difference, an increasing trend of CDK4 expressions by cordycepin from 50 to 100 μmol/L at 12‐hour treatment could be observed (Figure 3B). However, there was a decreasing trend of CDK4 expressions by cordycepin from 0 to 100 μmol/L after 24‐hour exposure. But, treatments with 500 and 1000 μmol/L cordycepin for 24 hours did induce CDK4 expressions in MA‐10 cells (Figure 3B). Overall, the expressions of FoxO3a, FoxO1, FoxO4, P15, and P27 could be down‐regulated by cordycepin, indicating that cordycepin affects MA‐10 cell survival by blocking cell cycle progression in G1/S phase to induce apoptosis.

### 3.3 Cordycepin‐induced apoptosis in MA‐10 cells by activating PERK/EIF2α signaling pathways

**Figure 4:**
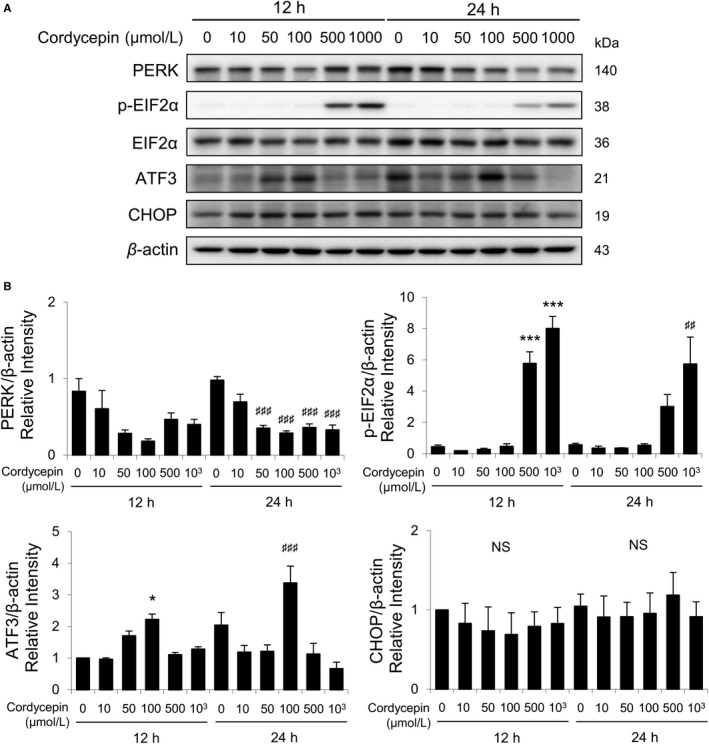
Cordycepin‐induced apoptosis of MA‐10 cells by activating PERK/EIF2α signaling pathways. A, Western blot analysis for the expression of total and phosphorylated EIF2α, total PERK, ATF3, and CHOP in MA‐10 cells treated with 0, 10, 50, 100, 500, or 1000 μmol/L cordycepin for 12 or 24 h, respectively. B, Quantification of bar graphs shows the IOD of EIF2α, phosphor‐EIF2α, PERK, ATF3, and CHOP, which were normalized with β‐actin (43 kDa) in each lane, respectively. Each data point represents the mean ± SEM of three separate experiments. The *P*‐values were calculated using one‐way ANOVA with Tukey's multiple comparisons post‐tests; * or ^#^ *P* < 0.05, ** or ^##^ *P* < 0.01, and *** or ^###^ *P* < 0.001 vs the control group (0 μmol/L cordycepin) at 12 or 24 h, respectively. ANOVA, analysis of variance; IOD, integrated optical density

Studies have demonstrated that misfolded proteins could prompt ER stress to restore protein homeostasis. If stress is prolonged, apoptotic cell death succeeds.^27^, ^28^, ^29^ To further study whether cordycepin would regulate ER stress pathways inducing apoptosis in MA‐10 cells, ER stress‐related proteins, such as PERK, EIF2α, p‐EIF2α, ATF3, CHOP, ATF6β, IRE1α, cleaved XBP1, and cleaved caspase‐12, in cordycepin‐treated MA‐10 cells were analyzed by Western blotting (Figure 4A). Results showed that expression of PERK gradually decreased by cordycepin treatments (0‐100 μmol/L); however, expression of PERK rebounded by treatments of 500 and 1000 μmol/L cordycepin for 12 hours (Figure 4B). Interestingly, expression of PERK significantly decreased by cordycepin (50‐1000 μmol/L) in 24‐hour treatment (Figure 4B). Expressions of p‐EIF2α were stimulated by 12‐ and 24‐hour treatments of 500 and 1000 μmol/L cordycepin, respectively (Figure 4B). Expression of ATF3 was stimulated by 100 μmol/L cordycepin after 12‐ and 24‐hour treatments (Figure 4B). The CHOP expression showed no significant change with cordycepin treatments for 12 and 24 hours (Figure 4B).

**Figure 5:**
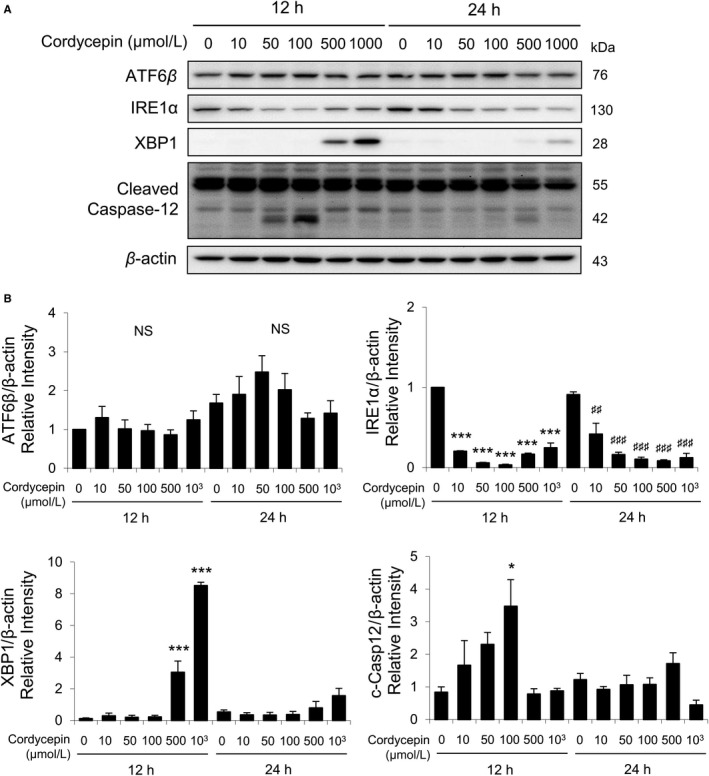
Cordycepin‐induced apoptosis of MA‐10 cells by activating ATF6 and IRE1 signaling pathways. A, Western blot analysis for the expression of total ATF6β, IRE1α, XBP1, total, and cleaved caspase‐12 in MA‐10 cells treated with 0, 10, 50, 100, 500, or 1000 μmol/L cordycepin for 12 or 24 h, respectively. B, Quantification of bar graphs shows that the IOD of ATF6β, IRE1α, XBP1, and cleaved caspase‐12 (c‐Casp12), which were normalized with β‐actin (43 kDa) in each lane, respectively. Each data point represents the mean ± SEM of three separate experiments. The *P*‐values were calculated using one‐way ANOVA with Tukey's multiple comparisons post‐tests; * or ^#^ *P* < 0.05, ** or ^##^ *P* < 0.01, and *** or ^###^ *P* < 0.001 vs the control group (0 μmol/L cordycepin) at 12 or 24 h, respectively. ANOVA, analysis of variance; IOD, integrated optical density

Total ATF6β, IRE1α, cleaved XBP1plus total and cleaved caspase‐12 were also detected by Western blotting with the treatments of cordycepin (0, 10, 50, 100, 500, and 1000 μmol/L) for 12 and 24 hours. Results showed that12‐ and 24‐hour cordycepin treatments did not affect the expressions of ATF6β (Figure 5A,B). Treatments with cordycepin (10‐1000 μmol/L) for 12 and 24 hours significantly decreased the expressions of IRE1α (Figure 5B). A known value of 1000 μmol/L cordycepin induced the maximal levels of XBP1 expression at 12 hours (Figure 5B). Furthermore, 50 and 100 μmol/L cordycepin induced the expressions of cleaved caspase‐12 at 12 hours (Figure 5B).

These results indicated that cordycepin could regulate ER stress pathways to induce apoptosis in MA‐10 cells.

### 3.4 Cordycepin affected cell cycle distribution differently in attached and suspended MA‐10 cells

**Figure 6:**
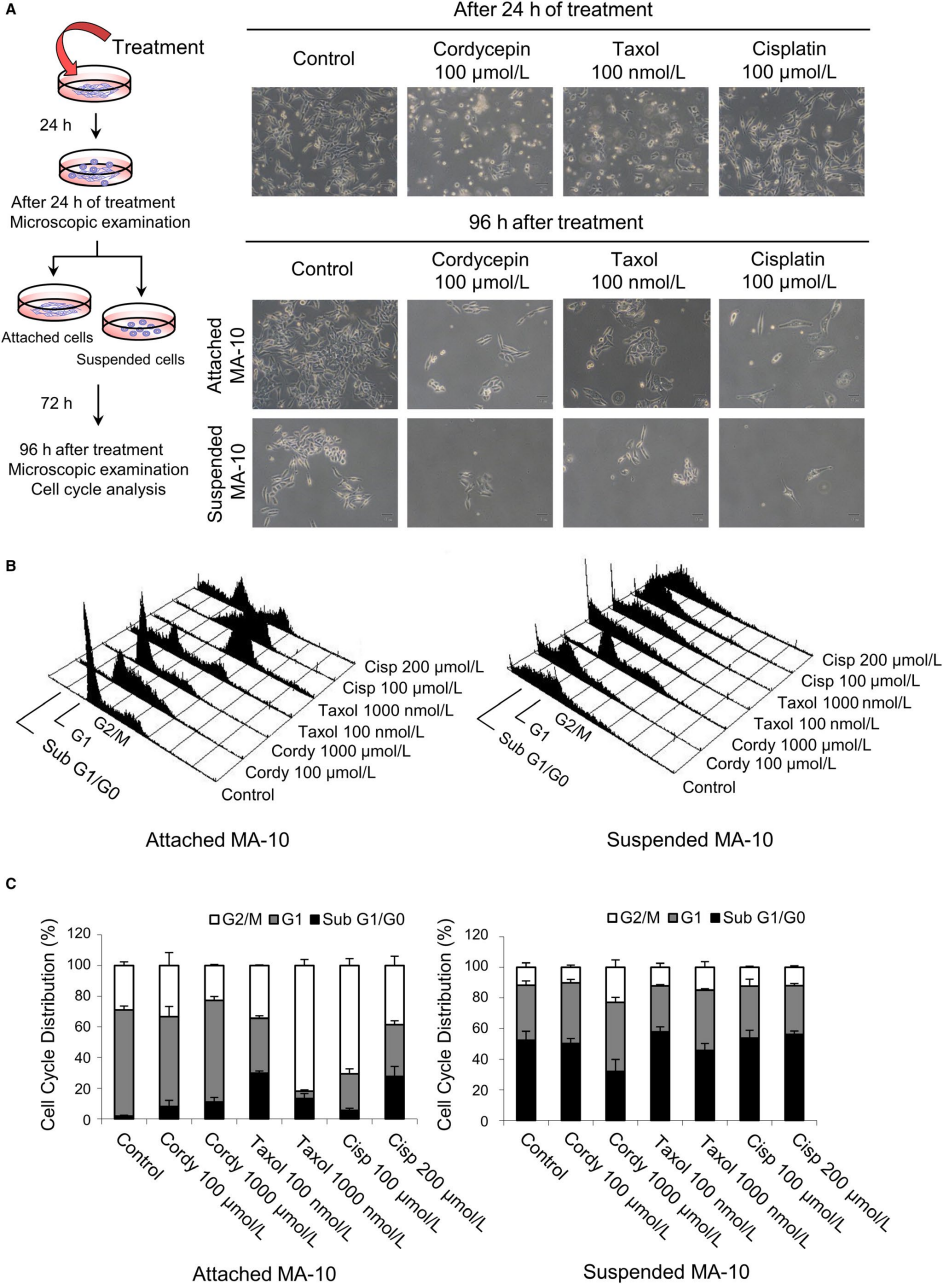
Cordycepin affected cell cycle distribution differently in attached and suspended MA‐10 cells. A, MA‐10 cells were treated with cordycepin (100 μmol/L), taxol (100 nM), cisplatin (100 μmol/L), or plain media (control) for 24 h. Suspended and attached MA‐10 cells were collected separately and then recultured for a further 72 h. Cell viability and morphological changes were assessed by light microscopy after 24 and 96 h of treatments. B, Representative flow cytometric histograms and (C) bar graphs show the cell cycle distributions of suspended and attached MA‐10 cells treated with cordycepin (100 and 1000 μmol/L), taxol (100 and1000 nmol/L), cisplatin (100 and 200 μmol/L), or plain media (control) for 24 h. Data represent mean ± SEM for four independent experiments. Cordy, cordycepin; Cisp, cisplatin

Cordycepin increased cell death by ER stress‐induced apoptosis in MA‐10 cells. However, we also found that some MA‐10 cells could survive after the cordycepin treatment, indicating the existence of drug resistance to cordycepin in MA‐10 cells. To investigate this issue further, suspended and attached MA‐10 cells were collected separately after 24 hours with 100 μmol/L cordycepin treatment, and recultured for 72 more hours (96 hours after cordycepin treatment) (Figure 6A, left panel). The cell viability and morphological changes were assessed at 24 and 96 hours after cordycepin treatments. Results showed that cordycepin, taxol, and cisplatin (positive control) induced MA‐10 cell death with reduced cell number compared to control after 24‐hour treatment (Figure 6A, right panel). In the additional 72 hours recultured experiment, MA‐10 cells from the attached portion had better survival rate with higher attached cell number as compared to the suspended portion among control, cordycepin‐, taxol‐, and cisplatin‐treated groups (Figure 6A, right panel). These results indicate that there were different populations in MA‐10 cells, and the cells from attached part had greater drug resistance to cordycepin.

To examine the possible mechanisms how drug resistance to cordycepin occurred, MA‐10 cells were treated with cordycepin (100 and 1000 μmol/L), taxol (100 and 1000 nmol/L), cisplatin (100 and 200 μmol/L), or plain media (control) for 24 hours, respectively. Cells were then stained with propidium iodide and analyzed by flow cytometry to investigate the changes among fractions of sub G1/G0, G1, and G2/M phases. Results showed that percentage of sub‐G1 phase induced by 100 and 1000 μmol/L cordycepin in attached MA‐10 cells is lower than 10% (Figure 6B,C), whereas the percentage of subG1 phase induced by 100 and 1000 μmol/L cordycepin in suspenedMA‐10 cells is higher than 30%, indicating the attached MA‐10 cells did have lower cell death rate related to drug‐resistance phenomenon. In addition, the percentages of G2/M and M phases in the attached MA‐10 cells are higher compared to the suspended MA‐10 cells. These phenomena were also observed in the taxol‐ and cisplatin‐treated groups (Figure 6B,C).

### 3.5 Cordycepin resistance in MA‐10 cells is mediated by activating AKT/mTOR and autophagy pathway and inhibiting the caspase signaling pathway

**Figure 7:**
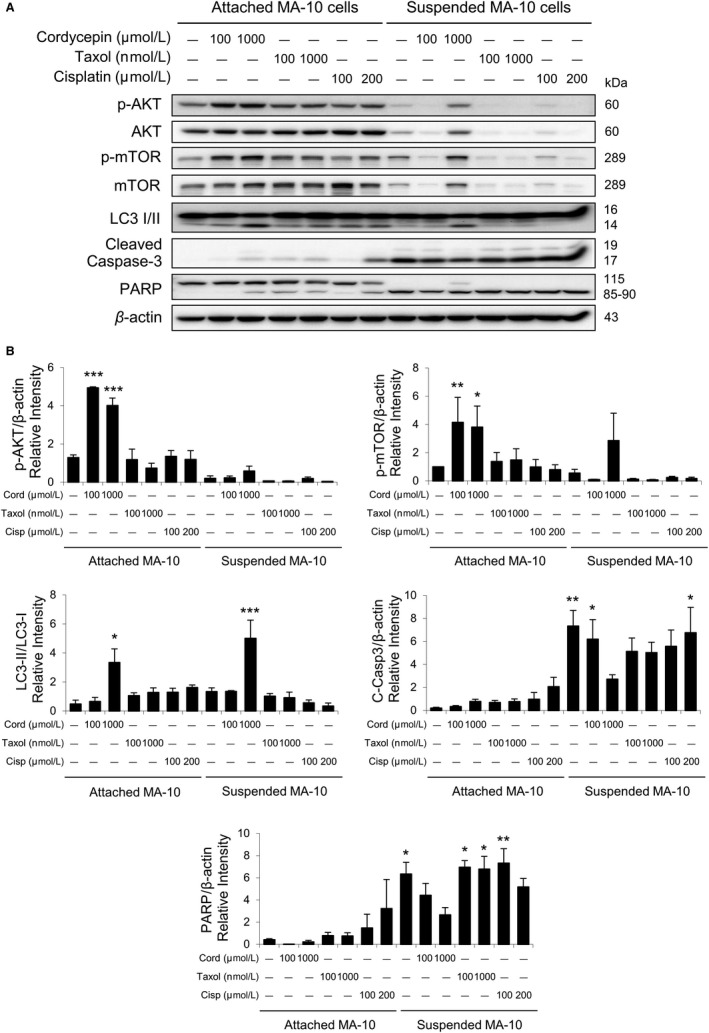
Cordycepin regulating AKT/mTOR signaling pathways in attached and suspended MA‐10 cells. A, MA‐10 cells were treated without or with cordycepin (Cord, 100 and 1000 μmol/L), taxol (100 and 1000 nmol/L), and cisplatin (Cisp, 100 and 200 μmol/L) for 24 h, respectively. MA‐10 cells were then collected from attached and suspended portions, and AKT, phosphor‐AKT, mTOR, phosphor‐mTOR, LC3 I/II, cleaved caspase‐3 (C‐Casp3), and PARP were detected by Western blotting. Immunoblot represents the observation from one single experiment, which was repeated at least three times. B, IOD of total AKT, phosphor‐AKT, mTOR, phosphor‐mTOR, LC3 I/II, cleaved caspase‐3, and PARP proteins were normalized with β‐actin in each lane, respectively. Each data point represents the mean ± SEM of three separate experiments. The *P*‐values were calculated using one‐way ANOVA with Tukey's multiple comparisons post‐tests; *P < 0.05, **P < 0.01, and ***P < 0.001 vs the control group (0 μmol/L cordycepin, taxol, and cisplatin), respectively. IOD, integrated optical density

Studies have demonstrated that Akt pathway is a pro‐survival pathway by activating pro‐survival signal cascades and inhibiting apoptotic signal cascades.^30^, ^31^, ^32^ To examine the underlying mechanisms of cordycepin resistance, the PI3KAkt/mTOR and related pathways for surviving in attached and suspended MA‐10 cells were carried out after 24‐hour cordycepin treatments. Results showed that the phosphorylation of Akt and mTOR in attached MA‐10 cells were induced more by cordycepin as compared to the suspended MA‐10 cells (Figure 7A,B).

Although the roles of autophagy in tumorigenesis have not been fully clarified, studies have pinpointed the autophagy for novel therapeutic targets in anticancer therapy due to the diverse metabolic status and necessity on stress responses between normal and cancer cells.^12^, ^33^ In the present study, expression of LC3 I/II was activated by 1000 μmol/L cordycepin in both attached and suspendedMA‐10 cells (Figure 7A,B). However, expressions of apoptotic makers, cleaved caspase‐3 and PARP, were much lower in attachedMA‐10 cells as compared to the suspended cells (Figure 7A,B).

These results demonstrated that pro‐survival AKT pathway and autophagy were activated, and pro‐apoptotic caspase pathway was reduced in attached MA‐10 cells, illustrating that drug resistance to cordycepin does exist in MA‐10 cells.

### 3.6 Cordycepin resistance in MA‐10 cells is mediated by activating MAPK signaling pathways

Studies have shown that MAPKs pathway is essential in the progression of cancer, which do control cell growth, migration, proliferation, and differentiation.^34^, ^35^ MAPKs (ERK1/2, JNK and p38) are protein‐serine/threonine kinases, which could be activated through phosphorylation, beginning with the induction of MAPK kinase kinases (MAP3Ks) to adjust cell fates.^36^

**Figure 8:**
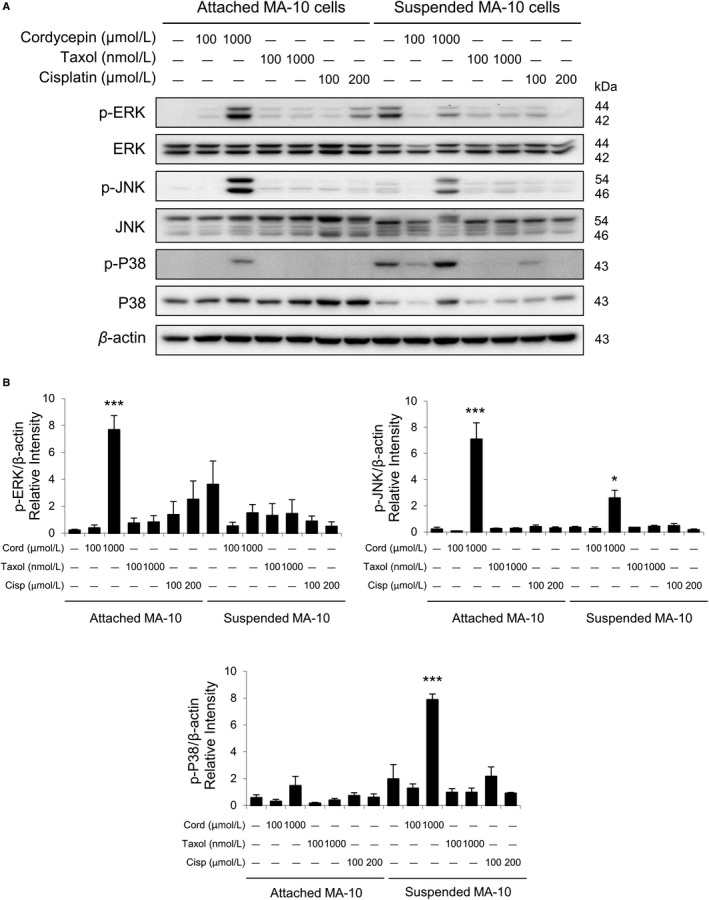
Cordycepin activating MAPK signaling pathways in attached and suspended MA‐10 cells. A, MA‐10 cells were treated without or with cordycepin (Cord, 100 and 1000 μmol/L), taxol (100 and 1000 nmol/L) and cisplatin (Cisp, 100 and 200 μmol/L) for 24 h, respectively. MA‐10 cells were then collected from attached and suspended portions, and ERK, phosphor‐ERK, JNK, phosphor‐JNK, p38, and phosphor‐p38 were detected by Western blotting. B, IOD of phosphor‐ERK, phosphor‐JNK, and phosphor‐p38 were normalized with β‐actin in each lane, respectively. Each data point represents the mean ± SEM of three separate experiments. The *P*‐values were calculated using one‐way ANOVA with Tukey's multiple comparisons post‐tests; *P < 0.05, ** *P* < 0.01, and ***P < 0.001 vs the control group (0 μmol/L cordycepin), respectively. IOD, integrated optical density

To investigate whether cordycepin could induce MAPK pathways for surviving related to drug resistance between attached and suspended MA‐10 cells, the expressions of p‐ERK, p‐JNK, and p‐P38 were analyzed by Western blotting after 24 hours of cordycepin treatments. Results showed that 1000 μmol/L cordycepin significantly stimulated the expressions of p‐ERK and p‐JNK in attached MA‐10 cells, which were higher than that in suspended MA‐10 cells (Figure 8A,B). In contrast, 1000 μmol/L cordycepin significantly stimulated the expression of p‐P38 in suspended MA‐10 cells, but not in attached MA‐10 cells (Figure 8A,B).

These results demonstrated that cordycepin could induce p‐ERK and p‐JNK expressions and reduce p‐P38 expression in attached MA‐10 cells to illustrate the possible mechanism upon drug resistance to cordycepin in MA‐10 cells.

## 4 DISCUSSION

**Figure 9:**
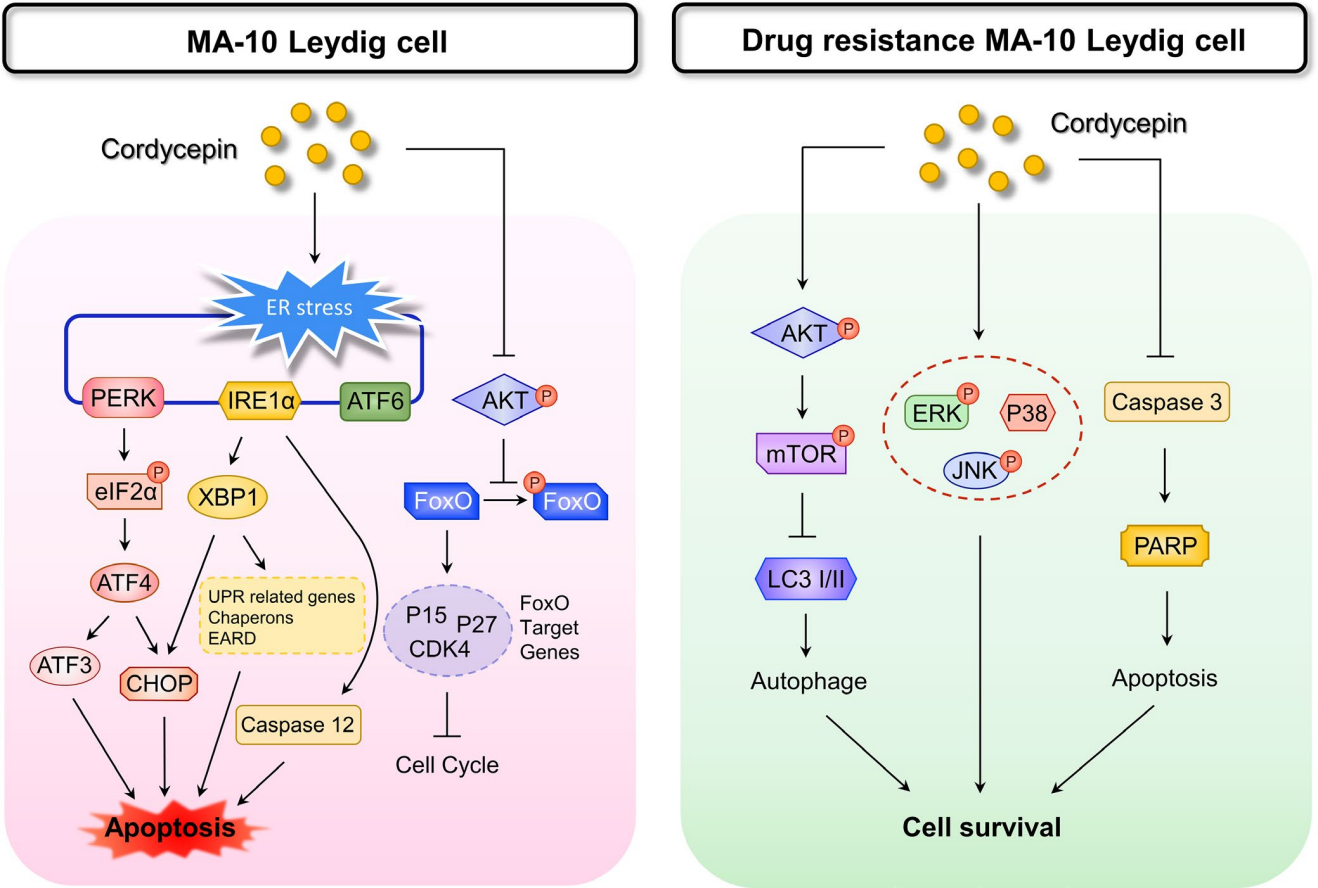
Summary scheme represents the possible pathways of drug sensitive and drug resistance to cordycepin in MA‐10 mouse Leydig tumor cells. Cordycepin reduced the activation of FoxO/P15/P27 signaling pathways and induced the PERK‐eIF2α (apoptotic) axis and the IRE1‐XBP1 (adaptive) UPR pathway. Additionally, activation of AKT and MAPK pathways could possibly result in drug resistance to cordycepin in MA‐10 cells

We have previously published that cordycepin reduced cell viability and induced apoptosis through activating caspases, inducing cell cycle arrest, regulating p38 MAPKs signaling, increasing ROS levels, and suppressing PI3K/AKT signaling in MA‐10 cells.^24^ In this study, microarray analysis results showed that genes related to cell cycle regulations and UPR/ER stress could possibly play important roles inducing MA‐10 cell apoptosis (Figures 1 and 2). We revealed that cordycepin could induce MA‐10 cell death by reducing FoxO/P15/P27/CDK4 pathways (Figure 3) to arrest cell cycle^24^ and inducing ER stress to cause UPR‐dependent apoptosis through activating the PERK/eIF2α/ATF3/CHOP (apoptosis) and the IRE1/XBP1 (protein refolding) pathways. We also found that there was a population of MA‐10 cells with cordycepin drug‐resistance phenomenon through activating Akt/mTOR, ERK, and JNK pathways and reducing the P38 pathway (Figure 9).

Cordycepin could affect cell cycle to cause apoptosis by UPR/ER stress with the changes among G2/M, G1, and subG1 phases between treatments in MA‐10 cell, especially subG1 phase with significant increase induced by cordycepin which did correlate to our previous study.^24^, ^37^ Many studies have shown that subG1 phase change related to DNA fragmentation is one of a good marker for apoptosis.^38^, ^39^ Thus, our results are parallel to their studies.

In the clustering analysis of mRNA expression, FoxO/P15/P27/CDK4, PERK/eIF2α/ATF3/CHOP (apoptotic), and the IRE1/XBP1signaling pathways were identified as central hubs of the apoptosis‐related interactive network in the cordycepin‐treated MA‐10 cells. Study has shown that forkhead box (FOX) transcription factors consist of 19 subfamilies sharing conserved DNA‐binding domain, the FOX domain, and the O subgroup comprises FoxO1, FoxO3, FoxO4, and FoxO6.^40^ Studies have illustrated that FoxO transcription factors function as signaling integrators among various transcriptional networks to maintain cell and tissue homeostasis in response to environmental hazards, and abnormal expression could play essential roles in cancerdevelopment.^40^, ^41^, ^42^ Our data showed that phosphor‐FoxO1, phosphor‐FoxO3a, and phosphor‐FoxO4 all decreased by cordycepin, except phosphor‐FoxO3a was stimulated by 500 and 1000 μmol/L cordycepin at 12‐hour treatment. Thus, cordycepin could down‐regulated phosphor‐FoxO1, phosphor‐FoxO3a, and phosphor‐FoxO4 to induce MA‐10 cell apoptosis. However, the high doses of cordycepin stimulate p‐FoxO3a to induce apoptosis in MA‐10 cells.

ER stress could trigger UPR to induce protein folding capacity and to decrease unfolded protein load.^43^ If ER stress is persistently extended, cell death would occur through apoptosis, which could be activated by PERK, ATF6, and IRE1α.^28^ In the present study, cordycepin at different doses and different time periods stimulated the expressions of UPR downstream proteins (PERK, EIF2α, ATF3, CHOP, ATF6β, XBP1, and cleaved caspase‐12) and reduced IRE1α in MA‐10 cells, demonstrating that cordycepin would modulate ER stress pathways to induce MA‐10 cell apoptosis. We have to notice that cordycepin at 500 and 1000 μmol/L did significantly induced PERK, EIF2α, and XBP1 expressions. Whether the up‐regulation of PERK, EIF2α, and XBP1 by high dosage of cordycepin is involved with drug resistance should be valuable to be further investigated.

We have found that some MA‐10 cells could survive with cordycepin treatment, indicating the drug resistance to cordycepin did exist in MA‐10 cells. Cell viability and morphological analysis related to drug resistance to cordycepin did show that in 72 hours of more recultured experiments, MA‐10 cells from the attached portion did have better survive rate with higher attached cell number as compared to the suspended portion among control groups. Studies have illustrated that the activation of PI3K‐AKT‐mTOR pathway and autophagy^44^, ^45^, ^46^ and the inactivation of pro‐apoptotic caspase pathway^47^, ^48^ are involved in the therapeutic resistance for cancers. We observed that pro‐survival AKT pathway and autophagy were activated and pro‐apoptotic caspase pathway was reduced in attached portions of MA‐10 cells, illustrating the possible mechanism of drug resistance to cordycepin in MA‐10 cells. Thus, our observations are not unparalleled.

MAPKs pathway is essential in cancer progression, which could control cell differentiation, proliferation, and migration.^34^, ^35^ Studies have shown that the activation of MAPKs pathways is involved to the therapeutic resistance for various cancers.^49^, ^50^ We have found that cordycepin did induce the expressions of phosphor‐ERK and phosphor‐JNK in attached portion, but not in suspended portion, of MA‐10 cells. However, cordycepin at 1000 μmol/L significantly stimulated more expression of phosphor‐P38 in the suspended portion compared to the attached portion of MA‐10 cells. In this study, our observation that 100 uM cordycepin did not increase phosphor‐p38 expression in the attached portion of MA‐10 cells for survival, is different from our previous study that cordycepin stimulated phosphor‐p38 expression to induce apoptosis in MA‐10 cells.^24^ These findings imply that there are different populations in MA‐10 cells, and some of them (from the attached portion after 24 hr‐cordycepin treatment) could be resistant to cordycepin. Drug‐resistance to cordycepin in MA‐10 cells could be the upregulation of phosphor‐ERK and ‐JNK with down‐regulation of phosphor‐p38 in the attached portion of MA‐10 cells.

In conclusion, FoxO and ER stress signaling pathways are required for pro‐apoptotic phenomenon in MA‐10 cell death following cordycepin treatment, suggesting a potential therapeutic application in treating testicular cancer. However, drug resistance to cordycepin could occur, which could be the activation of AKT and MAPK pathways, especially up‐regulation of ERK and JNK and down‐regulation of p38, in MA‐10 cells.

## CONFLICT OF INTEREST

The authors have no conflict of interests.

## AUTHOR CONTRIBUTIONS

MMC and BSP contributed equally to this work. BMH conceived the project. MMC, BSP, and BMH designed the experiments, carried out the experiments, interpreted results, and wrote the manuscript. JYW helped with data interpretation and the manuscript editing.

## Data Availability

The data that support the findings of this study are available from the corresponding author upon reasonable request.
