# Supplementary material for: Cordycepin‐induced unfolded protein response‐dependent cell death, and AKT/MAPK‐mediated drug resistance in mouse testicular tumor cells
Source: Cancer Med. 2019 May 30;8(8):3949–64. doi: 10.1002/cam4.2285 (PMC6639181; doi:10.1002/cam4.2285)
Supplement: Supplementary file 1 [file CAM4-8-3949-s001.docx]

**Table S1.** Chemicals and materials used in this study.

| Chemical / Material | Manufacturer^a^ | Catalogue No. |
| --- | --- | --- |
| 2-ME (2-Mercaptoethanol) | Alfa Aesar / Thermo Fisher Scientific | A15890 |
| Acrylamide/Bis-acrylamide, 30% solution | Sigma-Aldrich | A3574-100ML |
| Bovine serum albumin (BSA) | UniRegion BioTech | UR-BSA001-100G |
| Cisplatin | Sigma-Aldrich | P4394-25MG |
| Citric Acid | J. T. Baker | 0122-01 |
| Cordycepin | Sigma-Aldrich | C3394-25MG |
| DMSO | Sigma-Aldrich | D4540-500ML |
| ECL  (Immunobilon® chemiluminescent HRP substrate) detection kit | Millipore | WBKLS0500 |
| EDTA | Sigma-Aldrich | E5134-100G |
| EGTA | Merk | L808635342 |
| Ethanol | J.T.Baker | 8006-05 |
| Fetal bovine serum (FBS) | Gibco | 10437-028 |
| Glycine | J.T Baker | 4059-06 |
| HEPES [4-(2-hydroxyethyl)-1-piperazinee-thanesulfonic acid] | Sigma-Aldrich | H4034-100G |
| Hydrochloric acid (HCl) | Merck | K49418617738 |
| Methanol | DUKSAN | 62 |
| MTT [3-(4,5-dimethylthiazol-2-yl)-2,5-diphenyltetrazolium bromide] | Sigma-Aldrich | M5655 |
| Paraformaldehyde (PFA) | Merk | 1,04005.1000 |
| [Penicillin/Streptomycin](https://www.google.com.tw/search?q=Penicillin/Streptomycin&spell=1&sa=X&ved=0ahUKEwi7jNTY--rcAhVb_GEKHZwQC9AQkeECCCMoAA) | Simply | CC502-0100 |
| Potassium chloride (KCl) | Sigma-Aldrich | P5405-250G |

**Table S1.** Chemicals and materials used in this study (continued).

| Chemical / Material | Manufacturer^a^ | Catalogue No. |
| --- | --- | --- |
| Potassium dihydrogen phosphate (KH_2_PO_4_) | Sigma-Aldrich | P5655-100G |
| Propidium iodide (PI) | Sigma-Aldrich | P4170-25MG |
| Protease Inhibitor Cocktail | Sigma-Aldrich | P8340-1ML |
| Protein Asssay Dye | BioRad Laboratories | 5000006 |
| Sodium bicarbonate (NaHCO_3_) | Sigma-Aldrich | S5761-500G |
| Disodium hydrogen phosphate (Na_2_HPO_4_) | Sigma-Aldrich | S5136-100G |
| Sodium azide | Sigma-Aldrich | S2002-100G |
| Sodium chloride (NaCl) | Sigma-Aldrich | S6191-1KG |
| Sodium chloride (NaCl) | J.T Baker | 3624-05 |
| Sodium dodecyl sulfate (SDS) | SERVA | 20765.03 |
| Sodium hydroxide (NaOH) | Sigma-Aldrich | S2770 |
| Sodium orthovanadate | Sigma-Aldrich | S6508 |
| Sodium pyrophosphate | Riedel-de Haën | 30411 |
| Taxol | Sigma-Aldrich | T7402-5MG |
| TBE buffer (5x) | UniRegion Bio Tech | UR-TBEL |
| Tris base | J.T. Baker | 4109-06 |
| Tris HCl | J.T. Baker | 4103-02 |
| Triton X-100 (for cell lysate) | GERBU | 2000 |
| Trypsin-EDTA (0.5%, 10x) | Gibco / Thermo Fisher Scientific | 15400-054 |
| Tween 20 | PanReac AppliChem | 123412-1611 |

**Table S1.** Chemicals and materials used in this study (continued).

| Chemical / Material | Manufacturer^a^ | Catalogue No. |
| --- | --- | --- |
| Waymouth MB 752/1 medium | Sigma-Aldrich | W1625-1L |
| PVDF membrane (Polyvinylidence difluoride membrane; 0.45 micron pore size) | PALL Life Science | BSP0161 |

^a^ The headquarters locations of companies: **BD** (**Becton, Dickinson and Company),** Franklin Lakes, NJ, USA; **BioTnA**, Kaohsiung, Taiwan; **BDH laboratory Supplies**, Kampala, Uganda; **DUKSAN**, Ansan City, Kyunggi, Korea; **GeneDirex** , Taichung, Taiwan; **Gibco**, Grand Island, NY, USA; **GERBU Biotechnik GmbH,** Heidelberg, Germany; **J.T.Baker/Fisher Scientific UK Ltd**, Loughborough, UK; **Merck**, Darmstadt, Germany; **Millipore**, Billerica, MA, USA; **EMD Millipore**, Billerica, MA, USA; **PALL Corporation,** Port Washington, NY, USA; **PanReac AppliChem**, Iselin, NJ, USA; **PerkinElmer**, Waltham, Massachusetts, USA; **PEPROTech**, Rocky Hill, NJ, USA; **Riedel-de Haën/Honeywell**, Morristown, NJ, USA; **Thermo Fisher Scientific Inc.**, Waltham, Massachusetts, USA;  **UniRegion Biotech**, Taipei, Taiwan.
