# Supplementary material for: Cordycepin‐induced unfolded protein response‐dependent cell death, and AKT/MAPK‐mediated drug resistance in mouse testicular tumor cells
Source: Cancer Med. 2019 May 30;8(8):3949–64. doi: 10.1002/cam4.2285 (PMC6639181; doi:10.1002/cam4.2285)
Supplement: Supplementary file 2 [file CAM4-8-3949-s002.docx]

**Table S2.** Antibodies used in this study.

| Antibody | Manufacturer^a^ | Catalogue No. | Species/Clonality | Application^b^ |
| --- | --- | --- | --- | --- |
| **Primary Antibody** | | | | |
| AKT | Cell Signaling | #9272 | rabbit/polyclonal | WB |
| Phosphor-AKT (Ser473) | Cell Signaling | #9271 | rabbit/polyclonal | WB |
| ATF3 | Santa Cruz Biotechnology | sc-22978 | rabbit/polyclonal | WB |
| ATF4 | Cell Signaling | #11815 | rabbit/monoclonal | WB |
| ATF6β | ABGENT | AP5101b | rabbit/polyclonal | WB |
| β-actin | Sigma-Aldrich | A5441 | mouse/monoclonal | WB |
| CDK4 | Santa Cruz Biotechnology | sc-23896 | mouse/monoclonal | WB |
| CHOP | Santa Cruz Biotechnology | sc-7351 | mouse/monoclonal | WB |
| Cleaved-Caspase 3 | Cell Signaling, | #9661 | rabbit/polyclonal | WB |
| Cleaved-Caspase 12 | Cell Signaling | #2202 | rabbit/polyclonal | WB |
| ERK1/2 | Cell Signaling | #9102 | rabbit/polyclonal | WB |
| Phosphor-ERK1/2  (Thr202/Tyr204) | Cell Signaling | #9101 | rabbit/polyclonal | WB |
| Phosphor-FoxO1(Thr24) / FoxO3α (Thr32) / FoxO4(Thr28) | Cell Signaling | #2599 | rabbit/monoclonal | WB |
| IRE1α | Cell Signaling | #3294 | rabbit/polyclonal | WB |
| Phosphor-IRE1α (S724) | Abcam | Ab48187 | rabbit/polyclonal | WB |
| IRE2α | ABGENT | AP13469a | rabbit/polyclonal | WB |
| Phosphor-EIF2α (Ser51) | Cell Signaling | #3398 | rabbit/polyclonal | WB |
| JNK | Cell Signaling | #9252 | rabbit/polyclonal | WB |
| Phosphor-JNK (Thr183/Tyr185) | Cell Signaling | #9251 | rabbit/polyclonal | WB |
| LC3 I/II | Cell Signaling | #4108 | rabbit/polyclonal | WB |

**Table S2.** Antibodies used in this study (continued).

| Antibody | Manufacturer ^a^ | Catalogue No. | Species/Clonality | Application ^b^ |
| --- | --- | --- | --- | --- |
| **Primary Antibody** | | | | |
| p38 | Cell Signaling | #9212 | rabbit/polyclonal | WB |
| Phosphor-p38  (Thr180/Tyr182) | Cell Signaling | #9215 | rabbit/polyclonal | WB |
| IgG | Jackson ImmunoResearch | 005-000-003 | goat | Negative control |
|  |  | 011-000-003 | rabbit |  |
| mTOR | Cell Signaling | #2983 | rabbit/polyclonal | WB |
| Phosphor-mTOR (Ser2448) | Cell Signaling | #2971 | rabbit/polyclonal | WB |
| PARP | Cell Signaling | #9532 | rabbit/monoclonal | WB |
| PERK | Cell Signaling | #3192 | rabbit/monoclonal | WB |
| p15 | Santa Cruz Biotechnology | sc-612 | rabbit/polyclonal | WB |
| p27 | Cell Signaling | #3698 | rabbit/polyclonal | WB |
| XBP1 | ABGENT | AP5088C | rabbit/polyclonal | WB |
| **Secondary Antibody** ^b^ | | | | |
| Anti-mouse IgG HRP-conjugated | PerkinElmer | NEF82200-1EA | Goat | WB |
| Anti-rabbit IgG HRP-coniugated | PerkinElmer | NEF81200-1EA | Goat | WB |

^a^ The headquarters locations of companies: **Abcam**, Cambridge, UK; **ABGENT**, San Diego, USA; **Cell Signaling**, Beverly, MA, USA; **GeneTex**, Irvine, CA, USA; **Invitrogen**, Waltham, MA, USA; **Jackson ImmunoResearch**, West Grove, PA, USA; **Oncogene Science**, San Diego, CA, USA; **PerkinElmer**, Waltham, Massachusetts, USA; **Proteintech**, Rosemont, IL, USA ; **Santa Cruz Biotechnology**, Santa Cruz, CA, USA; **Sigma-Aldrich**, St. Louis, MO, USA.

^b^ WB, western blot analysis; HRP, horseradish peroxidase.
